# Burden of the Cardiovascular Diseases in Central Asia

**DOI:** 10.5195/cajgh.2018.321

**Published:** 2018-08-08

**Authors:** Altyn Aringazina, Tleuberdi Kuandikov, Viktor Arkhipov

**Affiliations:** 1 Department of Population Health and Social Sciences, Kazakhstan School of Public Health, Medical University, Almaty, Republic of Kazakhstan; 2 Department of Anesthesiology and Intensive Care of National Scientific Centre of Surgery, Almaty, Republic of Kazakhstan

**Keywords:** Cardiovascular Diseases, Epidemiology, Central Asia

## Abstract

Cardiovascular diseases (CVD) are now the number one cause of death in low- and middle-income countries, including those in Central Asia (CA). Low- and middle-income countries (LMICs) bear a disproportionate and growing burden of CVD, which constitutes a challenge to national development. CVD account for more than 43% of cases of disability and 9.0% of cases of temporary disability in many developing countries. The high burden of CVD oftentimes results from insufficient preventive care and a lack of education about the prevention and treatment of these diseases. The rapidly growing burden of CVD and other major non-communicable diseases (NCDs) is a global public health threat, especially in Central Asia. Information on cardiovascular risk factors, including hypertension, diabetes, tobacco use, and alcohol use, is traditionally obtained from studies conducted in Europe and North America, which limits our understanding of these factors in Central Asia. In this review, we collected all published information on CVD in Central Asia from 2000 to 2015, which included the websites of the Ministries of Health, the World Health Organization, PubMed, and other published sources. This narrative review describes CVD burden, stroke incidence, and common CVD risk factors in the five post-Soviet countries of Central Asia (Kazakshstan, Kyrgyzstan, Tajikistan, Turkmenistan, and Uzbekistan).

Central Asia (CA) is a region consisting of five former Soviet republics, including Kazakhstan (18 million), Kyrgyzstan (5.7 million), Tajikistan (8.0 million), Turkmenistan (5.2 million), and Uzbekistan (30 million), with a total population of approximately 66 million inhabitants^1^. Cardiovascular diseases (CVD) are the number one cause of death in low- and middle-income countries (LMICs), such as those in CA^2^. The high burden of these conditions oftentimes results from insufficient preventive care and lack of education about the prevention and treatment of these diseases. The rapidly growing burden of CVD and other major non-communicable diseases (NCD) is a major public health challenge in CA^3^.

It is projected that CA countries will experience an increase in the total number of deaths due to NCDs by 2020^1^. The World Health Organization (WHO) estimated that 17.5 million people died of CVD in 2012, accounting for 46% of all NCD deaths^3^. Of these deaths, an estimated 7.4 million were due to coronary heart disease, and 6.7 million were due to stroke and hypertension^3^. More than 80% of these deaths occurred in low and middle-income countries, and CVD are now the number one cause of death in CA^4^. Worldwide, nearly 25.7 million people had strokes in 2013 (71% was ischemic stroke), of which 6.5 million died (51% was ischemic stroke). The mortality from CVD in CA countries is generally higher than that in Europe^5^.

Information on cardiovascular risk factors, including hypertension, diabetes, tobacco use, and alcohol use are traditionally derived from studies conducted in Europe and North America. For this study, we reviewed information from a variety of sources on CVD in Central Asia from 2000 to 2015 from published sources, including the websites of the Ministries of Health and the World Health Organization, as well as PubMed, and other published sources. The purpose of this review is to describe and analyze data on CVD in CA for the following reasons. First, the policy makers of CA countries face enormous difficulties in reforming healthcare in extremely unfavorable economic conditions, and during major domestic and political transformations. To help these countries rebuild their health systems, policymakers need access to reliable information about their own health systems and local public health indicators. Second, CA countries have an interest in discovering initiatives that can be successfully adapted from abroad. It should be noted that most data regarding CA healthcare systems is limited or not available for public access. To our knowledge, reliable scientific and statistical publications on CVD in Turkmenistan were not available as of 2017. Thus, this review does not include the Republic of Turkmenistan.

## Republic of Kazakhstan

In 2014, the overall mortality rate from CVD in Kazakhstan was 232.4 per 100,000; in 2015 it was 219 per 100,000^6^. Regionally, the highest rate of CVD was reported in the Karaganda region with 368.1 deaths per 100,000 in 2015^6^. In rural areas, mortality figures were significantly lower than in urban areas. Among urban residents in 2014, the general mortality rate from CVD was 238.3 per 100,000, while in rural areas it was 162.2 per 100,000. Kazakhstan has the third highest death rate from ischemic stroke among the countries of the former Soviet Union^7^.

During the period between 2011 and 2015, a national screening program for CVD and diabetes was introduced in Kazakhstan. Screening of CVD in Kazakhstan takes place every 2 years through free medical care in all district clinics for men and women aged 18, 25, 30, 35, and 40–64 years who have not been diagnosed with heart disease or diabetes. During the screening, patients are given a questionnaire assessing risk factors and had their height, weight, blood pressure, cholesterol, and blood sugar measured^6^. Those with a high risk of cardiovascular mortality received referrals for further care. From 2011 to 2015, over 7.5 million adults in Kazakhstan were screened for CVDs. As a result, more than 600,000 cases of heart disease were identified (7.9% of those screened)^8^.

Arterial hypertension is one of the most common diseases in Kazakhstan and poses a serious challenge to public health. Between 2009 and 2013 the prevalence of hypertension significantly increased from 10,778 to 13,392 per 100,000^8^, resulting in 24.3% of adults in Kazakhstan having hypertension in 2013^8^,^9^. Mortality rates related to hypertension increased as well and now rank first among causes of death. Approximately 40% of the deaths were observed in working age group (20–64 years), 64% of which were males^6^. To curb this epidemic, the State Health System established a screening program for the early detection of CVD and its risk factors^9^–^11^. Most of the screened patients had not previously received any treatment for their conditions^12^.

According to the WHO Report on the Global Tobacco Epidemic (2013), the prevalence of tobacco use among the adult population of Kazakhstan (aged 15–65 years) was 29.8% (48.0% for males and 12.1% for females)^13^. The consumption of smokeless tobacco among young people was 3.0% (3.4% for males and 2.7% for females)^11^.

In comparison, the results of five national studies on monitoring and surveillance of tobacco consumption in Kazakhstan showed a small decrease in smoking over the past 14 years^13^. In 1998, the prevalence of smoking among young people over the age of 11 was 28.0%, 49.8% among men and 12.2% among women. In 2012 this figure was 26.5%, 41.5% among men and 11.0% among women^13^. In 2006, Kazakhstan joined the Framework Convention on Tobacco Control, and committed itself to implementing measures to protect the public from tobacco smoke^14^. The Chief Sanitary Doctor of the Ministry of Health of the Republic of Kazakhstan adopted ban on smoking hookah in public places in March of 2013^15^. The government supports initiatives aimed at the prevention of non-communicable diseases by adoption of a healthy lifestyle in the population.

Kazakhstan is 34^th^ in the world in terms of alcohol consumption (10.3 liters of alcohol consumed per capita per year) and is the largest consumer of alcohol among the CA countries^16^. From 2008–2012, the volume of alcoholic beverages sold in Kazakhstan increased by 9.6% from 862 million liters to 944.4 million liters. From 2013 to 2017, the growth in sales of alcoholic beverages in Kazakhstan was projected to average 1.7% per year. In 2017, estimated domestic sales of alcoholic products were expected to reach 1.028 million liters. According to the 2003 World Health Survey (total sample size of 2894; 1170 males and 1724 females), the mean value (in grams) of pure alcohol consumed per day among drinkers was 2.9 (total), 4.2 (males) and 2.1 (females). While alcohol consumption in general carries additional health risks unrelated to CVD, extremely high levels of alcohol consumption may increase the risk of CVD in an individual.

In Kazakhstan, stroke treatment is becoming a priority with 41 world-class stroke centers opening in different regions of the country in the past few years, and 30 more centers projected to open before 2020^17^. Under the Ministry of Health and Social Development of Kazakhstan, a coordinating council was formed to implement an integrated model of health care delivery for socially significant NCDs^17^. The Ministry of Health focused its efforts on conditions like acute myocardial infarctions, acute cerebrovascular accidents, malignant neoplasms, trauma, and pregnancy complications^18^. Although many health status measures show that Kazakhstan is ahead of most nations in the region, it continues to lag behind other countries with similarly sized economies on several important health indicators^7^.

## Republic of Kyrgyzstan

Among countries incuded in the WHO European Region report, Kyrgyzstan has the highest premature mortality rate from CVD, the second-highest death rate from cerebrovascular disease, and the third-highest death rate from ischemic heart disease^19^. In Kyrgyzstan, mortality from stroke is much higher, and ischemic heart disease is moderately lower than in other post-Soviet countries. Life expectancy at birth in Kyrgyzstan is 75 years for women and 67 years for men^19^. NCDs are estimated to account for 80% of all deaths; with half of these deaths attributed to CVD. The probability of dying between the ages of 30 and 70 years from the main NCDs (ischemic stroke and ischemic heart disease) is 28%^20^,^21^. Nevertheless, there is a downward trend for premature mortality from NCDs (largely driven by reductions in CVD mortality), and projections suggest that Kyrgyzstan will reach the global NCD target of a 25% reduction in mortality by 2025^21^. In recent years, there has been a reduction in the total mortality rate due to CVD, with a decrease from 331.3 in 2012 to 300.9 per 100,000 in 2015^22^. Mortality rate reduction was also observed for stroke and acute myocardial infarction in men and women, although the reduction in the latter mortality rate was larger in women than men^21^. In contrast, according to the National Statistics, mortality for all ages due to ischemic stroke has increased^23^.

Apart from the differences in risk factor prevalence, other reasons for higher mortality among men relate to lack of awareness of the signs, symptoms, and consequences of raised blood pressure and underutilization of health-care services^24^.

Kyrgyzstan has a relatively low gross domestic product compared to other post-Soviet countries and was categorized as a low-income country by the World Bank until 2014^25^. However, the general government expenditure on health as a percentage of total government expenditure is relatively high compared to similar countries, with total expenditure on health close to that of 12 post-Soviet median-income countries^20^. The trends in select causes of death are similar to those in other post-Soviet countries, but are slower to change, which can be linked to the health-system’s limited capacity for early detection and treatment^20^. The prevalence of cardiovascular risk factors is high; these include diet, high blood pressure, and tobacco use^21^. A recent WHO study describing the nutritional composition of the foods sold in Bishkek, Kyrgyzstan, found that the amounts of trans-fatty acids and salt in common foods are extremely high compared to the developed contries^26^.

High blood pressure was always on the top of the list of health problems identified during health assessments in Kyrgyzstan population. It was the third most common disease for women, the second most common for men, and exerts a large burden on populations living in poverty^20^,^22^. Government initiatives have been formed to focus on screening programs to improve people’s awareness of hypertension. Since 2011, an annual “hypertension week” has been held, during which Village Health Committees (VHCs) provided blood pressure screenings, and explained the dangers of elevated blood pressure and CVD^27^. The Community Action for Health (CAH) program 2014^27^ has contributed to a significant improvements in the early detection and management of hypertension, and the number of people undergoing screening is increasing annually. Since 2011, a total of 1.75 million people were screened for elevated blood pressure, comprising about half of the adult population of Kyrgyzstan^23^. The CAH program had a significant nationwide impact on hypertension awareness and control. According to WHO calculations from the nationally representative Integrated Household Survey, hypertension awareness increased from 27% in 2007 to 45% in 2015^28^. The increase was greater in rural areas, where VHCs work. As a result, a large urban–rural gap in awareness of hypertension status noted in 2007 had disappeared by 2015^28^. Compliance with anti-hypertensive medication also improved during this period. The proportion of people with elevated blood pressure who reported having taken their medication in the past 24 hours was 33% in 2015, in contrast to 14% in 2007^28^.

Nicotine and alcohol consumption are important factors contributing to CVD mortality in Kyrgyzstan. The prevalence of current tobacco smoking among Kyrgyzstan population aged 15 years and older in 2013 was 3.7% for women, and 50.5% for men^21^. The total annual per capita alcohol consumption among people aged 15 years and older was 4.3 liters of pure alcohol per year in 2011^21^.

Additionally, obesity is a problem in Kyrgyzstan. In 2014, the percentage of overweight males and females aged 18 years and above was 45.2% and 49.1% respectively 21. The WHO STEPS NCD survey in 2013 reported that 42.9% of adults aged 25–64 years had elevated blood pressure (similar frequency in males and females) and 23.6% had an elevated total cholesterol levels (more common in females than in males)^29^. Almost one in five (17.4%) adults were identified as being at high cardiovascular risk, i.e. the probability of their having a cardiovascular event or death in the next ten years was 30% or more. Over a third of adults aged 25–64 years had three or more cardiovascular risk factors; this rate was higher among men (39.5%) and older age groups^29^.

In Kyrgyzstan, there is an established political and legislative framework for the prevention and control of CVD. There are a national strategy to combat NCDs for 2013–2020, which recently passed a mid-term evaluation, and the National Health Reform Program “Den Sooluk” for 2012–2016^22^, which prioritizes the health of the cardiovascular system. The regulatory and fiscal framework for tobacco control is underdeveloped, but its scope can be expanded, and its enforcement can be strengthened^22^.

Funding of the CVD Action Plan includes implementation of a package of essential NCD (PEN) interventions at the primary health-care level (PEN protocols) as well as an article that provides free access to screening^22^. “Den Sooluk” was originally planned to end in 2016 but because of a delayed start, the Government of Kyrgyzstan and donor partners agreed to extend it until the end 2018. A mid-term review of “Den Sooluk” reported that 25% of the 96 indicators have been achieved or exceeded as part of the main goal of reducing the burden of CVD’s^30^.

## Republic of Tajikistan

The epidemiological situation shows that CVD are the leading cause of death in Tajikistan^31^. Between 1990 and 2010, Tajikistan had an increase in the burden of NCDs, especially coronary heart disease and stroke^31^. In addition, it is estimated that about 40% of the total population is overweight and 9% is obese, which suggests a low level of physical activity and unhealthy dietary habits^32^. There is limited access to emergency medical services for the acute myocardial infarction. The most important services are provided at the level of the central district hospitals (prescribing aspirin, beta blockers, and angiotensin converting enzyme inhibitors), but access to thrombolytic therapy is limited^33^. For the treatment of stroke, obsolete methods are used that are not based on evidence-based medicine^34^. The country lacks evidence-based clinical guidelines for the management of stroke patients^34^. During 2005–2011, CVD mortality increased from 63 to 67 cases per 100,000, or approximately from 46.8% to 48.82% of the total death rate^35^.

Hypertension is among the major risk factors for CVD. Many Tajik women suffer from hypertension without knowing it; hypertension is often termed the ‘silent killer’ because of the lack of warning signs or symptoms. In the 2012, Tajikistan Demographic and Health Survey (TjDHS)^32^ respondents completed several questions to determine their history of hypertension, including whether they have ever been told by a doctor or other health worker that they had high blood pressure and, if so, whether they had been told that on two or more occasions. If surveyed women reported that on one or more occasions they were told that they had high blood pressure, they were asked additional questions on actions they were taking at the time of the survey to lower their blood pressure. Overall, the TjDHS^32^ results indicate that 12% of women aged 15–49 reported having been told by a doctor or other health worker that their blood pressure was high. Seventy-eight percent of women with high blood pressure reported that they were diagnosed with hypertension on two or more occasions. More than eight in ten of those women were taking medication to control their blood pressure. A significant percentage of women reported not taking other measures to lower their blood pressure; only 46% were cutting back on salt in their diet, 39% were controlling or losing weight, and 29% were exercising. As expected, the prevalence of women with high blood pressure increased with age, from 3% of women aged 15–19 to 29% of women aged 45–49. Also, being overweight (BMI >25) was strongly correlated with high blood pressure. The proportion of women with high blood pressure was slightly higher in rural women compared to urban women (30% and 21% respectively). This can be explained by the fact that a relatively large proportion of women are receiving medical care in urban health facilities^32^.

Tajikistan made progress in the fight against tobacco by amending the Law on Restricting the Use of Tobacco Products in early 2011^36^, and ratifying the WHO Framework Convention on Tobacco Control (WHO FCTC)^37^ which was launched on September 19, 2013^38^–^40^. Despite these amendments, the laws on tobacco control still need improvements. In particular, it is necessary to clarify and explain the terminology associated with what is considered to be tobacco products^38^. Nevertheless, youth smoking monitoring and control are better in Tajikistan compared to other CA countries. The Global Youth Tobacco Survey (GYTS), an inter-country study conducted in CA, was conducted in Tajikistan in 2014^41^ and showed that in Tajikistan only 5.9% of students used any tobacco products (6.8% of men and 2.8% of women), while the average results in other CA countries were reported to be higher (9.6%)^41^.

The level of alcohol consumption in Tajikistan is relatively low^42^. The total annual per capita alcohol consumption among people aged 15 years and above was 4.3 liters of pure alcohol per year^42^. According to the recent survey of rural population, only 12.2% of men and 0.1% of women consumed alcohol^38^. Among the urban population, 39% of men and 6.7% of women consumed alcohol^38^. Advertising alcohol is prohibited in Tajikistan, although it can still be found in retail stores.

Tajikistan has made progress in promoting healthy eating and physical activity. In 2011, a governmental intersectoral working group was established to develop and implement the strategy for improving nutrition and physical activity, including an action plan for 2013–2020 based on WHO strategies^43^. The strategy defines priority areas, such as reducing consumption of salt, trans-fats, and sugar, and promotes exclusive breastfeeding, timely and appropriate supplementary nutrition, as well as a healthy diet and physical activity. Since 2004, Tajikistan has implemented a series of comprehensive measures to reform the health sector to address the problem of limited access to health services. The reforms envision changing the organization and provision of medical services by moving to the family medicine model and introducing appropriate financial mechanisms to reduce the level of out-of-pocket cash payments^44^–^46^. These efforts still face challenges, in particular, the need to expand the coverage of basic individual services, strengthen the tobacco control, increase the level of state funding, strengthen the coordination between providers, and improve the quality of medical services at the level of primary health care.

## Republic of Uzbekistan

Uzbekistan registers more than 1.5 million acute and chronic CVD cases on an annual basis, with more than half a million of them being newly diagnosed cases. Over the past 10 years, the level of primary and general morbidity in this population has increased^47^. Since 2003, the primary incidence rate has increased 1.4 times (from 1,291 to 1,759 per 100,000), and the general prevalence increased 1.2 times (from 4,672 to 5,503 per 100,000)^48^. Detection of the primary morbidity and registration of general morbidity from CVD among the adult population increased 1.3 and 1.1 times (7,154 and 6,053 per 100,000) respectively^48^. Morbidity from CVD among children (0–14 years) and teens (15–18 years old) has decreased 1.2 times (4,586 per 100,000) between 2003 and 2013^48^. Such a decrease among younger age groups is probably associated with a program on maternal and child health implemented in 1998. The increase in the level of primary and general morbidity from CVD among the adult population is probably due to the increase in life expectancy in the country from 67 to 73.1 years^48^. In addition, the measures taken in the Republic of Uzbekistan to improve the quality of primary health care, and the development of healthy lifestyles have increased the number of individuals receiving medical care^48^. The level of coverage by preventive examinations of certain population groups for the study period increased to 88–89% for adults and to 99–99.6% for children^49^. The level of access to doctors in polyclinics (primary care facilities) and rural medical stations increased 1.3 times when compared to 2003 and amounted to 9 visits per capita per year in 2013^49^. It is estimated that by 2020 the level of the primary and general morbidity from the CVD across the Republic will be 2,069 per 100,000 (60% increase) and 6,062 per 100,000 (30% increase) respectively^48^.

CVD traditionally have low-ranking positions on the list of morbidity causes, contributing only 6.8% all causes of morbidity in 2013. This may be explained by the relatively young population; 32% of population is under 18 years of age. However, in the structure of the causes of death in the country, they occupy a leading place, and in 2012 their share was 61.6% 48. Among all leading causes of the overall morbidity 40.4% is occupied by diseases characterized by an elevated blood pressure, including hypertension with target organ damage (14.2%), angina (7.7%), chronic ischemic disease heart disease (7.0%), cerebrovascular disease (4.1%), acute myocardial infarction (0.54%), and 5.9% by chronic rheumatic heart disease^48^.

A study aimed at hypertension, the women’s and men’s questionnaires for the Uzbekistan Health Examination Survey (UHES), was performed in 2002 with no more recent data available. Rates of hypertension among women aged 15–49 and men aged 15–59 were 7 to 8% respectively^50^. 74% of women had a blood pressure reading in the optimal range (< 120/80 mmHg) compared to 48% of men^50^. In general, rates of hypertension were positively associated with age, education, urban residence, and being overweight/obese. More hypertensive women than men were aware of their condition (62% versus 37%), and higher number of women than men managed their condition with medication (37% versus 10%)^50^.

In Uzbekistan, a study based on the WHO STEPS methodology was conducted between January and April of 2014^51^. This study suggested that the prevalence of smoking among adults (18–64 years) was 14.4% (26.8% of men and 1.4% of women)^51^. Smoking is prohibited in all enclosed public places except for designated smoking areas. However, there are no specially allocated funds to enforce this ban, nor is there a system for filing and considering citizens’ complaints about violations of the smoking ban^52^. In accordance with the Law on Advertising, adopted in 1998, which was subsequently amended^53^, and the law on restricting the distribution and consumption of alcohol and tobacco products adopted in 2011, certain types of direct and indirect advertising of tobacco products are banned. However, in the event of violation of these prohibitions, a penalty in the form of a fine is not enforced^52^.

According to the recent rural population survey, only 12.2% of men and 0.1% of women used alcohol^38^. These figures were 39% for men and 6.7% for women in urban populations^37^,^54^. The total annual per capita alcohol consumption among people aged 15 years and above was 4.6 liters of pure alcohol per year^51^. Prices on alcohol are quite low and may stimulate an increase in alcohol consumption.

## Conclusions

NCDs, and particularly CVD, present a challenge for the CA. Ischemic heart disease and stroke are major causes of premature mortality, and the prevalence of cardiovascular risk factors is high within the CA population. Some of the regional governments have committed themselves to tackling the problem, as evidenced by the policy framework and some of the measures already in place. In CA countries stroke and ischemic heart disease are more prominent among NCDs. This is most likely due to a higher prevalence of CVD risk factors in CA countries. Reduction in salt consumption in CA countries is important for the reduction of CVD, especially for stroke. The high prevalence of elevated blood pressure is of great concern due to the fact that some with elevated blood pressure may be unaware of their condition. Such persons should be followed up and advised to utilize existing primary and secondary prevention opportunities. Secondly, those who were previously diagnosed with high blood pressure and were not effectively treated need to be followed up with by health care providers.

Prevention of smoking is also an important strategy for reducing CVD in most CA countries, especially for men. Smoking increases the risk of developing CVD by approximately 30%^55^. The STEPS survey will provide invaluable information needed to inform policy and planning. Tobacco smoking is an important modifiable behavioral risk factor in CA. Second hand smoking is something that has been rarely evaluated in CA and needs to be considered in future research.

Heavy alcohol consumption remains an important risk factor for global burden of CVD^56^. Nearly all the data on humans exploring the relationship between alcohol consumption and CVD risk, including some indications of potential CVD benefits associated with low-to-moderate alcohol consumption, are derived from epidemiologic studies. Therefore, because there are no randomized controlled trials, health care professionals should not recommend alcohol consumption as a primary or secondary lifestyle intervention. Instead, clinicians should continue to recommend strategies such as a healthy diet and exercise. For example, certain levels of alcohol consumption that lower risk for CHD may increase it for other CV conditions, such as stroke. In addition, data from studies using new research methods, including Mendelian randomization, suggest that the relationship between low-to-moderate alcohol consumption and cardioprotection merits more critical appraisal^56^.

The prevalence of obesity is also increasing in the region, potentially leading to the increased prevalence of diabetes, impaired glucose control, and metabolic syndrome. Since these conditions influence cardiovascular health, we will focus on these conditions in our future research. As of 2017, there were no major studies focusing on these problems in the region. We believe that the problem of obesity and diabetes deserves close attention from the regional institutions, WHO, and other international organizations.
